# Mutual Effects of Components of Protective Films Applied on Steel in Octadecylamine and 1,2,3-Benzotriazole Vapors

**DOI:** 10.3390/ma14237181

**Published:** 2021-11-25

**Authors:** Andrey Y. Luchkin, Olga A. Goncharova, Nina P. Andreeva, Vadim E. Kasatkin, Sergey S. Vesely, Yurii I. Kuznetsov, Nikolay N. Andreev

**Affiliations:** A.N. Frumkin Institute of Physical Chemistry and Electrochemistry, Russian Academy of Sciences, Leninskii pr. 31, 119071 Moscow, Russia; skay54@yandex.ru (A.Y.L.); andrnin@mail.ru (N.P.A.); vadim_kasatkin@mail.ru (V.E.K.); sergei57@mail.ru (S.S.V.); yukuzn@gmail.com (Y.I.K.); n.andreev@mail.ru (N.N.A.)

**Keywords:** carbon steel, EIS, ellipsometry, atmospheric corrosion, passive films, passivity, corrosion inhibitors

## Abstract

In this work, we used a combination of corrosion, electrochemical, and physical methods to determine the properties of nanoscale films obtained by treatment with octadecylamine (ODA), benzotriazole (BTA) vapors, and their mixtures at elevated temperatures. The mixture of ODA + BTA surpasses its components in protective aftereffect, but an analysis of their mutual effects shows that there is antagonism between them. Electrochemical impedance spectroscopy data indicate that the protection of steel by a mixture of ODA + BTA and its components is characterized by a mixed blocking activation mechanism. The processing of steel in hot vapors of the ODA + BTA mixture leads to hydrophobization of the surface and super-hydrophobization if a polymodal surface is created on the steel before processing in vapors.

## 1. Introduction

The use of vapor phase inhibitors is a reliable and cost-effective way to protect metals from atmospheric corrosion [1,2]. There are two types of vapor phase inhibitors, namely, volatile corrosion inhibitors and chamber inhibitors (CIN).

Volatile corrosion inhibitors were developed in the middle of the 20th century and are now widely used in the industry. The theoretical and practical aspects of metal protection by these inhibitors are covered in many publications [1,2,3,4,5,6,7,8,9,10]. 

Along with the obvious advantages, the vapor phase protection of metals with volatile inhibitors has a number of serious limitations. The main one is that the space to be protected must be sealed for the entire protection period. The reason is that volatile corrosion inhibitors form adsorption films that provide the anticorrosive effect are in dynamic equilibrium with the environment inside that space. Molecules of a volatile inhibitor undergo continuous desorption and evaporation from the surface of the metal. Desorption of the volatile inhibitor is replenished by its adsorption from the atmosphere saturated with its vapors. If the airtightness of the space being protected is broken, the volatile inhibitor evaporates from it, the equilibrium is violated, and the protective adsorption films gradually degrade. After a certain time, which is dependent on the strength of inhibitor adsorption and its vapor pressure, corrosion initiation in a corrosive environment may occur. At the same time, the low vapor pressure of the inhibitor favors its anticorrosive aftereffect and protection efficiency [4].

However, inhibitors with a saturated vapor pressure below 10^−^^5^ mmHg are almost unsuitable for the vapor phase protection of metals in its traditional meaning [4]. If an attempt is made to use such an inhibitor as a volatile corrosion inhibitor for preservation, its molecules do not have sufficient time to reach the metal surface in an amount that would completely protect it before corrosion causes noticeable damage to the metal item. The vapor pressure limitation makes it unfeasible to perform vapor phase protection by numerous compounds that have both high inhibitory activity and environmental safety. 

The restrictions described above are lifted if the chamber variant of vapor phase protection is used. This fundamentally new preservation method was developed at the Institute of Physical Chemistry and Electrochemistry of the Russian Academy of Sciences approximately five years ago and has been intensely developing in recent years [11,12,13,14,15]. The main idea of the method is as follows. The vapor pressure of compounds increases with temperature (*T*). By increasing the temperature of exposure of a metal together with a low-volatile inhibitor in a closed space, one can significantly reduce the time (from up to a few hours down to several minutes) required to form protective adsorption films from the vapor phase. Such processing is called chamber treatment (CT) since passivation takes place in a sealed chamber. After the metal has cooled down, such films can protect metal products from atmospheric corrosion due to their aftereffect for the period of transportation and storage. In such a case, airtight packaging of metal items together with an inhibitor is not required. 

The new method significantly expands the scope of inhibitors suitable for vapor phase protection by including low-volatile compounds. Moreover, chamber protection is highly efficient and environmentally safe. A very small amount of the active compound is consumed in this process [11,14]. It does not result in continuous wastes, i.e., wash waters, depleted or polluted solutions, or spent packaging materials containing inhibitors. It is important that a CIN is only placed in the working space for the duration of the treatment and does not directly contact the personnel performing the anti-corrosion treatment or the environment. 

Like in the case of volatile corrosion inhibitors, versatile CIN formulations capable of protecting a few metals, rather than just one, have the best prospects for practical use. A mixture of octadecylamine (ODA) and 1,2,3-benzotriazole (BTA) is an example of such a formulation. 

Note that ODA and BTA are low-toxicity compounds that have good thermal resistance and possess low vapor pressure (below 10^−^^4^ Torr) at room temperature. However, at 80 °C it increases by a few orders of magnitude and reaches a range from 10^−^^2^–10^−^^1^ mmHg. At 140 °C, the vapor pressure of both compounds amounts to 1–5 mmHg [16]. Such properties indicate that these CIN have the potential to provide a good protective aftereffect (PAE) and, at the same time, the capability to quickly form adsorption films in the course of CT.

To confirm this assumption, two related studies were undertaken dealing with the determination of the optimal conditions for the CT of steel, copper, and brass with ODA, BTA, and an ODA + BTA mixture and the specifics of the protection of these metals. This article reports the first of these studies that deals with steel protection.

## 2. Materials and Methods

### 2.1. Samples

Samples and electrodes used in the study were made of St3ps. The steel composition complies with the corresponding standard [17]. All the reagents used in this work, including ODA and BTA, were of “pure” grade. The specimens intended for corrosion tests were flat and 30 × 50 × 3 mm^3^ in size. Each specimen had a hole to mount them in cells and chambers. Cylindrical electrodes had a diameter of 1 cm, giving a working area of 0.785 cm^2^. A threaded hole for the mounting rod was provided in one of their flat ends. The samples were embedded in Teflon casings to prevent the interaction of the side surfaces with the electrolyte. The lower flat end of a cylinder was used as the working electrode. 

Before each experiment, the working surfaces of the samples and electrodes were polished with emery paper of various grades (100–14 Microns), cleaned with acetone, washed with water, and then dried. 

In the studies on the metal surface super-hydrophobization with the CIN, polymodal roughness was created on the samples by laser surface texturing [18]. A nanosecond laser (wavelength 1064 nm) was used for the treatment. The pulse duration was from 50–200 ns, the average nominal power was from 15–20 W, the beam linear velocity was from 50–150 mm/s, the pulse frequency was from 20–90 kHz, and the pattern density was from 10–20 lines mm^−^^1^.

### 2.2. Accelerated Corrosion Tests in Conditions of 100% Humidity with Intermittent Moisture Condensation on Samples

Samples and electrodes were prepared as described above and then mounted in sealable glass vessels with 0.6 L capacity containing a weighed portion of a CIN (0.5 g) or without it. A SNOL 50/350 drying oven was heated to a required temperature (80, 100, 120, or 140 °C) and then the vessels were placed therein. After exposure in the oven was completed, the vessels were retrieved and allowed to cool to ambient temperature. The samples and electrodes were then removed from the vessels containing the inhibitor vapors and kept for 24 h under ambient conditions.

The protective aftereffect of the surface layers formed in the course of chamber treatment was determined at 100% relative humidity with daily moisture condensation. Metal samples were hung on Nylon filaments to the lids of sealed glass cells in such a way that prevented them from contact with each other and the cell walls. The volume of each cell was 0.6 L. A 0.1 L portion of hot water with a temperature of 50 °C was added to each cell, thus, causing vigorous condensation of moisture on the samples. Every 24 h, water that cooled down was replaced with hot water. For two days from the beginning of the tests, the samples were visually examined every hour through the transparent cell walls. After that period, they were examined every 6 h. The time (*τ*) until the corrosion damage was detected on the metal surface was recorded. 

### 2.3. Voltammetric Experiments

The protective properties of the CIN and mechanisms of protection were studied by recording polarization curves (voltammetric experiments) and electrochemical impedance spectra of steel samples that underwent treatment with the CIN in various modes. The experiments were performed using an electrochemical set-up comprising a versatile IPC-Pro MF potentiostat with an FRA module (made in RF). The measurements were performed in a three-electrode glass cell (50 mL) with divided electrode spaces. A platinum wire was used as the auxiliary electrode. The potentials (*E*) were measured versus a saturated Ag/AgCl reference electrode and converted to the standard hydrogen scale. Borate buffer solution (pH 7.36) with 0.001 M NaCl was used in the experiments.

Before recording a polarization curve, a metal sample treated with a CIN in some of the modes was placed in a cell and kept in the electrolyte for 15 min until its corrosion potential (*E_cor_*) reached a steady value. Potentiodynamic polarization curves were recorded from *E_cor_* in the anodic direction at 0.2 mV/s. The performance of a CIN was estimated by two values: the pitting potential (*E_pit_*) and breakdown potential (*E_br_*). The potential where the first current oscillations appeared on the polarization curves was taken as *E_pit_*. In the absence of current oscillations, *E_pit_* was considered to be equal to *E_br_*. *E_br_* was determined as the potential where the anodic current density (*j*) reached 2 μA/cm^2^.

### 2.4. Electrochemical Impedance Spectroscopy

**Figure 1:**
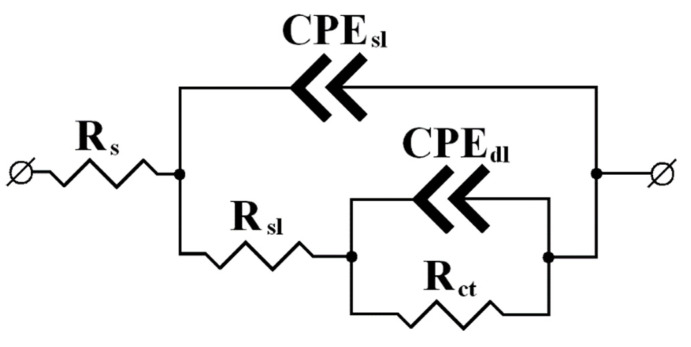
Equivalent circuit used to fit impedance data.

Impedance measurements were carried out under conditions similar to those used in the polarization experiments, in the same cell, and with similar electrodes. The measurements were carried out in the potentiostatic mode by applying a constant potential component equal to *E*_cor_ and a harmonic AC signal (amplitude = 10 mV) with frequencies from 100,000 to 0.1 Hz. The parameters of the equivalent circuit that adequately simulated the experimental impedance spectra were computed using the DCS program [19]. A modified Mansfeld circuit [20,21], where the capacitive elements were replaced with constant phase elements (CPE), was used in the simulations. This circuit type is widely used to simulate corrosion and the electrochemical behavior of metals with porous coatings and/or films (Figure 1) [22,23]:

In this circuit, *R_s_* designates the electrolyte resistance between the test sample and the reference electrode capillary. It is dependent on the solution conductivity, the distance between the sample surface and the capillary and does not affect the electrode processes. *R_sl_* is the total of the resistances of the oxide/hydroxide and adsorption surface layers, and *R_ct_* is the charge transfer resistance of the Faraday reaction that determines the corrosion process kinetics. *CPE_sl_* is a constant phase element that characterizes the capacitance of the metal–electrolyte interface and mainly depends on the structure of the surface layers. *CPE_dl_* is a constant phase element reflecting the capacitance of the electric double layer in the Faraday reaction. 

Unlike the classical Mansfeld scheme, the use of *CPE* elements instead of capacitances makes it possible to better simulate experimental data and obtain additional information on the nature of the electrode processes.

The CPE impedance was described by Equation [24]:

Z*_CPE_* = *A*^−1^(jω)^−n^, (1)

where *A* is a coefficient (modulus); j is the imaginary unit; ω is the cyclic frequency; n is the phase factor. Depending on the phase factor, the *CPE* element can be interpreted as a capacitor (*n* = 1), a Warburg diffusion element (*n* = 0.5), or a resistor (*n* = 0). With regard to the selected scheme, the value of the phase factor differing from unity can be used to judge how perfect the simulated capacitance is, how homogeneous its plates are, and how diffuse the charge distribution is. 

The extent of steel electrode protection was calculated as follows:

Z = (*R_CT_* − *R_TT_*)/*R_TT_* × 100% (2)

where *R_CT_* is the total of active resistances *R_ct_* and *R_sl_* in the equivalent circuit for a steel sample that underwent chamber treatment with the corresponding inhibitor. Similarly, *R_TT_* is the total of *R_ct_* and *R_sl_* for a “blank test” where the metal underwent only thermal treatment (TT).

### 2.5. Ellipsometry

The thicknesses (*d*) of films formed on the metal upon CT were determined using a Gartner manual ellipsometer with light beam modulation and an advanced light detector. An LSM-S-111-10-NNP25 solid-state laser with diode pumping and a wavelength of 540 nm served as the radiation source. The changes in ellipsometric angles Δ and ψ were measured during the experiment. These values were compared to the values calculated using the Ellipsometry Calculation Spread Sheet program (E. Kondoh, https://www.ccn.yamanashi.ac.jp/~kondoh/ellips_e.html, accessed on 3 March 2021) for various refraction indices and surface layer thicknesses. The layer thicknesses were varied to find the best fit between the calculated and experimental Δ and ψ values. The three-layer approximation model (metal-oxide layer adsorption film) was used to process the data.

### 2.6. Contact Angle Measurement

Contact angles (*θ*) were estimated using 2 μL droplets. A droplet was placed on the surface of a sample and a picture was taken by a digital camera equipped with a DCM 300 eyepiece. The *θ* value in the image was determined using a protractor available in the PicPick digital editor.

### 2.7. Outdoor Corrosion Tests

The PAE of surface layers was estimated in outdoor tests at the Moscow Corrosion Station. Samples that underwent CT were exposed on a rack under a shelter. They were examined once a week and the time when corrosion damage was detected (*τ_prot_*) was recorded. All the tests were conducted in three to five independent runs. We report the mean values.

## 3. Results and Discussion

### 3.1. Accelerated Corrosion Tests 

The protective performance of a CIN on St3ps steel was estimated under daily moisture condensation conditions in two series of experiments. In one of them, we determined the optimal CT temperature (*T_CT_*), while in the other one, its optimal duration (*τ_CT_*). In the first series of experiments, the adsorption films on the metal were created in the *T* range from 80–140 °C. The *τ_CT_* was 1 h in these experiments. 

The first corrosion damage (rust) on steel St3 that was not heat-treated was detected on the samples in 1.0 ± 0.5 h under the specified test conditions (Table 1). 

After steel chamber treatment at 80 °C, the inhibitors demonstrated almost no protective effects. An increase in *T_CT_* to 100 °C had practically no effect on the PAE of the adsorption films formed in BTA vapors. However, the protective properties of ODA and its mixture with BTA increased significantly under these conditions. The *τ_prot_* of the metal was 72 h (ODA) and 120 h (ODA + BTA mixture). 

Chamber treatment at 120 °C increased in the PAE of BTA adsorption films only slightly. However, the protective ability of ODA and ODA + BTA increased noticeably. After treatment with these CIN, the first indications of corrosion appeared on the samples after 120 or 168 h of steel exposure in the corrosive environment, respectively. 

A further increase in *T_CT_* (up to 140 °C) resulted in a decrease in the PAE of all the CIN studied. Chamber treatment with vapors of BTA, ODA, or their mixture provided steel protection for 2, 48, or 144 h, respectively. The possible reasons for the existence of a maximum on the temperature plot of the protective effect of CIN have been discussed elsewhere [14,15]. Most likely, the increase in the protective properties of the chamber inhibitors with an increase in the CT temperature results from an increase in their vapor pressure, and hence, stronger adsorption of the inhibitor on the metal. In turn, the downward branch is due to the well-known fact that adsorption decreases as the adsorbent’s temperature rises. Thus, 120 °C was found to be the optimal temperature of steel chamber treatment with the mixed CIN. 

The data presented above allow us to draw some conclusions about the mutual effects of ODA and BTA. Let us consider this phenomenon in more detail. 

According to a study by Ekilik and Chikov [25] relying on the formal theory of the action of corrosion inhibitors [26], the mutual effect of the components of an inhibitor mixture is determined by the ratio of the corrosion inhibition coefficients (γ) provided by the mixture (γ_mix_) and the product of the γ values of the components. It is important for our study, so let us repeat the main provisions. 

Let there be inhibitors “1” and “2” that are individually characterized by inhibition coefficients γ_1_ and γ_2_, respectively, in a corrosive medium of interest. Let the rate of general corrosion in this medium be K_0_ in the absence of inhibitors. Inhibitor “1” provides corrosion inhibition by a factor of γ_1_ to reach a value of K_1_:

K_1_ = K_0_/γ_1_ (3)

If γ_2_ does not depend on the presence of inhibitor “1” in the system, i.e., there are no interactions between the inhibitors, the addition of inhibitor “2” to the system will further reduce the corrosion rate by a factor of γ_2_. Then the corrosion rate in a system containing both inhibitors “1” and “2” (K_mix_) is:

K_mix_ = K_1_/γ_2_ (4)

Substitution of K_1_ from Equation (4) to this expression gives for the binary mixture:

K_mix_ = K_0_/(γ_1_γ_2_) (5)

It follows from Equation (6) that:

K_0_/K_mix_ = γ_1_γ_2_ (6)

or, taking into account that K_0_/K_mix_ = γ_mix_:

γ_mix_ = γ_1_γ_2_ (7)

Hence, if the addition of inhibitor “1” to the system slows down corrosion, for example, by a factor of 2, while the addition of inhibitor “2” slows it down additionally by a factor of 3, then their mixture will slow down corrosion by a factor of 6 if no mutual strengthening or weakening of the protective action occurs. 

Equation (8) is a criterion for the independence of the protective action of inhibitors of general corrosion of metals in binary mixtures. It was derived for acid corrosion but is also useful for neutral environments [27]. If γ_mix_ > γ_1_γ_2_, synergism is observed, i.e., the ability of inhibitors “1” and “2” to enhance the protective effects of each other. In contrast, if γ_mix_ < γ_1_γ_2_, this indicates that antagonism is observed, and the protective effect decreases. 

Let us develop the concept mentioned above [26] by adapting it to systems where the protective properties of inhibitors are estimated through *τ_prot_*. In such a case, Equation (8) takes the form:

*τ*_*prot, mix*_ = *τ*_*prot*,1_*τ*_*prot*,2_/*τ*_*prot*,0_ (8)

Let us consider a system where *τ_prot_*, i.e., the full protection period, is the criterion of inhibitor efficiency. Let us analyze the mutual effect of the components of a binary mixture of inhibitors “1” and “2” for this system. Let the first corrosion damage on the metal become visually noticeable upon transition of a metal with mass *M* on area *s* to an oxide-hydroxide form. Then the rate of the corrosion process that determines the formation of the first corrosion damage on the metal in the absence of inhibitors can be expressed as:

K_0_ = *M*/(*s τ_prot,_*_0_) (9)

It should be noted that both general and local corrosion may be concerned. 

Similarly, for inhibitor “1” that provides metal protection for a period of *τ_prot,_*_1_, inhibitor “2” whose metal protection period is *τ_prot,_*_2_, and a mixture of inhibitors “1” and “2” (*τ_prot,mix_*), the following expressions are valid:

K_1_ = *M*/(*s τ_prot,_*_1_) (10)

K_2_ = *M*/(*s τ_prot,_*_2_) (11)

and

K_mix_ = *M/(s τ_prot,mix_*) (12)

By definition, the coefficient of metal corrosion inhibition by inhibitor “1” can be expressed as:

γ_1_ = K_0_/K_1_ = [*M*/(*s τ_prot,0_*)]/[*M*/(*s τ_prot,1_*)] = *τ_prot,1_*/*τ_prot,0_* (13)

Similarly:

γ_2_ = K_0_/K_2_ = [*M*/(*s τ_prot,0_*)]/[*M*/(*s τ_prot,2_*)] = *τ_prot,2_*/*τ_prot,0_* (14)

and

γ_mix_ = K_0_/K_mix_ = [*M*/(*s τ_prot,0_*)]/[*M*/(*s τ_prot,mix_*)] = *τ_prot,mix_*/*τ_prot,0_* (15)

By substituting the expressions for γ into Equation (8) we obtain the following expression for a mixture of inhibitors “1” and “2” that do not interact with each other:

*τ_prot,mix_*/*τ*_*prot*,0_ = (*τ*_*prot*,1_/*τ*_*prot*,0_)(*τ*_*prot*,2_/*τ*_*prot*,0_) = *τ*_*prot*,1_*τ*_*prot*,2_/*τ*_*prot,*0_^2^ (16)

This expression can be transformed to Equation (8). 

Thus, expression (9) determines the lack of a mutual effect of the protective properties of inhibitors “1” and “2” expressed through the full metal protection time. The situation where *τ_prot,mix_ > τ_prot,_*_1_*τ_prot,_*_2_/*τ_prot,_*_0_ indicates that synergy exists, i.e., components “1” and “2” amplify the protective effects of each other, whereas if *τ_prot,mix_* < *τ_prot,_*_1_*τ_prot,_*_2_/*τ_prot,_*_0_, antagonism exists, i.e., the components mutually weaken the protective effects of each other. 

To estimate the mutual effects of components in mixed inhibitors more convenient, let us introduce a coefficient:

α = *τ_prot,mix_^meas^*/*τ_prot,mix_^calc^* (17)

where “meas” refers to a value measured experimentally, while “calc” refers to the result of a calculation by Formula (6). The value of α exceeding one indicates a synergism of protective effects. Moreover, the higher α, the more pronounced this interaction. 

A simple calculation shows that the coefficient α of the mutual effect of BTA and ODA in the tests described above was 0.23 and indicated an antagonism of the components. This antagonism may result from a decrease in the vapor pressure of the mixture due to the formation of a salt between a weak acid, BTA, and a base, ODA. 

At first glance, the antagonism of the protective action of ODA and BTA contradicts the fact that the efficiency of the mixed CIN is higher than that of its components. However, an analysis of the situation shows that there is no contradiction. This is very important for the creation of efficient CIN. Let us consider it in more detail in order to solve the following question: is synergism a prerequisite for creating formulations with a protective effect exceeding those of its components? 

It is easy to show that in the absence of any interactions, the protective properties of a mixture of inhibitors “1” and “2” are always higher than those of its components. Since compounds “1” and “2” are inhibitors, i.e., γ_1_ > 1 and γ_2_ > 1, the following expressions are valid:

γ_mix_ = γ_1_γ_2_ > γ_2_ (18)

and

γ_mix_ = γ_1_γ_2_ > γ_1_ (19)

Similarly, in the systems where the efficiency of inhibitors is expressed via *τ_prot_*, the following is true: *τ*_1_*/τ*_0_ > 1 and *τ*_2_*/τ*_0_ > 1. Then, for a binary mixture:

*τ_prot,mix_* = *τ*_*prot*,1_*τ*_*prot*,2_/*τ*_*prot*,0_
*> τ*_*prot*,2_ (20)

and

*τ_prot,mix_* = *τ*_*prot*,1_*τ*_*prot*,2_/*τ*_*prot*,0_
*> τ*_*prot*,1_ (21)

This means that the addition of two non-interacting inhibitors to a system is always accompanied by an increase in the protective ability in comparison with the individual components. This conclusion is quite obvious and not particularly useful in the practice of creating inhibitors for bulk solutions. In this case, the mixing of components is always accompanied by an increase in their total concentration in the electrolyte. In fact, inequalities (21) and (22) only indicate that as the total concentration of the inhibitor in a bulk electrolyte increases, the protective effect of the mixture increases. For example, if 5 g of inhibitor “1” and 5 g of inhibitor “2” are mixed together in a unit volume of a solution, then the total amount, i.e., 10 g of the mixture, will inhibit corrosion more efficiently than 5 g/l of any single inhibitor. 

The situation is different in the case of the chamber protection of metals where the protective properties of a CIN depend on the atmosphere composition. The content of chamber inhibitors in the atmosphere and their efficiency in a wide range of conditions is not dependent on their amount introduced into the chamber but is determined by their saturated vapor pressures in that chamber. Mixing of non-interacting CIN “1” and “2” increases the total vapor pressure of inhibitors in the system and the protection efficiency.

One more example: let us introduce 5 g of inhibitor “1” and 5 g of inhibitor “2” into a chamber. The mixture (10 g) will provide metal protection for a longer time than its components. However, the same protection will be provided by 5 g of the mixture, or 1 g of the mixture, etc. 

This is an answer to the question raised above. Synergism is desirable but not mandatory for the creation of mixed CIN whose efficiency exceeds that of the components. If there are no interactions between the components, their mixture will provide complete protection of a metal for a longer period than its components. Moreover, as shown by the data presented above, this is possible even if antagonism between the components is displayed. In fact, the calculated coefficient of the mutual effect of BTA and ODA (α = 0.23) indicates antagonism between the components. The reasons of this phenomenon are not entirely clear; however, it may be assumed that this antagonism is due to a decrease in the vapor pressure of the mixture due to the formation of a salt between a weak acid, BTA, and a base, ODA. 

The above results were obtained after steel chamber treatment for 1 h. According to the data in Table 2, this CT duration is sufficient to form adsorption films with the maximum PAE. Anyway, a 2- or 4-times longer CT of steel in vapors of ODA, BTA, or their mixture at the optimum temperature did not improve the corrosion protection of the samples. On the other hand, shortening the CT time to 20 min did decrease the protection efficiency. Surface layers that are in equilibrium at 120 °C cannot be formed on the metal in such a short time. Thus, the optimal *τ_CT_* of steel by the mixed CIN or by its components is 1 h.

The specific features of steel protection by the CIN in question were studied at *T*_CT_ = 120 °C and *τ_CT_* = 1 h. 

### 3.2. Voltammetric Experiments

**Figure 2:**
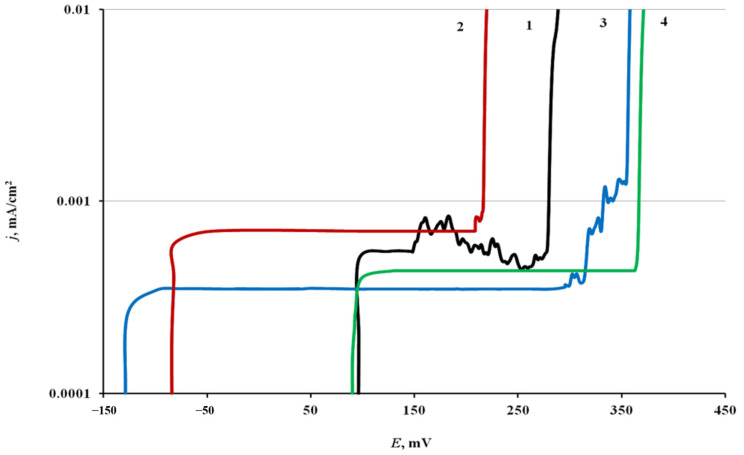
Polarization curves of St3 in borate buffer solution (pH 7.36) containing 0.001 M NaCl. 1: Without treatment with a CIN; 2: BTA; 3: ODA; 4: ODA + BTA.

The anodic potentiodynamic polarization curves of steel electrodes, both treated in CIN vapors and not subjected to CT, were typical of passive steel in a chloride-containing borate buffer solution (Figure 2 and Table 3). They have a wide passivity region, current oscillations associated with the initiation and repassivation of pits, and a sharp increase in the current density when *E*_br_ is reached. In some cases, there are no current oscillations on the polarization curves. 

The mean value of *E*_cor_ of a chamber-treated steel electrode in borate buffer was 0.095 V. Its anodic polarization did not result in noticeable changes in current up to *E* = 0.15 V. On reaching this potential, the oscillations that appeared on the polarization curves were due to pitting. This was confirmed by a visual inspection of the electrodes withdrawn from the electrolyte. Small pits on the electrode surface were visible through a magnifying glass. The further polarization of steel resulted in the breakdown of the passive film. The *j* values sharply increased at *E*_br_ = 0.27 V. In this case, dark dots with “scorch marks” on the metal were visible with a naked eye.

Chamber treatment of steel in BTA vapors shifted *E*_cor_ cathodically to −0.085 V. Anodic polarization of electrodes treated in this way resulted in passive film breakdown at *E*_br_ = 0.210 V. In this case, no current oscillations preceding the breakdown were observed on the polarization curves. 

After steel chamber treatment in ODA vapors, the stationary potential shifted to more negative values, *E*_cor_ = −0.130 V, than after treatment in BTA vapors. At *E* = 0.295 V, insignificant current oscillations appeared on the polarization curves, followed by passive film breakdown at *E*_br_ = 0.345 V.

Unlike BTA and ODA, the mixed CIN almost did not affect *E*_cor_, but it ennobled *E*_pit_ more strongly than the components (up to 0.36 V). Breakdown of the passive film occurred immediately, without current oscillations. 

Thus, the adsorption films of all the CIN in question stabilized the passive state of steel by increasing its resistance to local anionic depassivation. The ennoblement of *E*_pit_ caused by the mixed CIN exceeded the protective action of its components taken alone. 

### 3.3. Ellipsometry

The thickness of surface films often plays a decisive role in the choice of means and/or methods of the temporary protection of metal items. Moreover, information about the thickness of surface films plays a very important role in understanding the mechanism of the inhibitor effect. These considerations prompted us to perform the study described below. The thicknesses of adsorption layers that the mixed CIN and its components formed on steel under the optimal conditions of steel chamber treatment were estimated by ellipsometry. The measured thicknesses of the oxide and inhibitor films are shown in Table 4. 

These data indicate that in the initial state (after abrasive paper treatment and degreasing), the surface oxide films on steel are ca. 3 nm thick. One-hour treatment of steel at 120 °C resulted in a 5-fold thickening of the oxide. The addition of BTA into the chamber suppressed the growth of the oxide film so that its thickness did not exceed 4.5 nm. In this case, an adsorbed inhibitor layer 4 nm thick formed on the surface. ODA inhibited oxide growth less strongly than BTA and thicker adsorption films (up to 7 nm) formed. 

The ODA + BTA mixture nearly stopped the oxide film growth during heat treatment and caused the formation of a thinner adsorption layer than its components (*ca*. 2 nm). The comparison of these results with those of corrosion and electrochemical tests shows that the structure and regularity of passive films are more important for corrosion protection than their thickness. Thus, the results of ellipsometric measurements indicate that ultrathin nanosized films are formed on the surface during the CT of steel with the inhibitors studied. 

### 3.4. Electrochemical Impedance Spectroscopy

**Figure 3:**
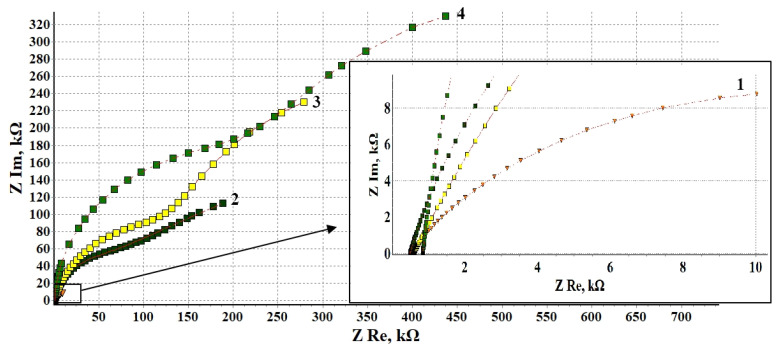
Nyquist diagrams of steel electrode after heat treatment in the absence of a CIN—1, and after CT: 2—BTA, 3—ODA, 4—ODA + BTA.

Additional information on the protective effect of the mixed CIN and its components on steel is provided by electrochemical impedance spectroscopy (EIS). The Nyquist diagrams of steel samples after heat treatment without an inhibitor and after treatment in CIN vapors are shown in Figure 3. 

All the Nyquist plots manifest two more or less distinct semicircular arcs, which makes it possible to apply a common equivalent circuit with two time constants in all the cases. The high-frequency (small) semicircle on the Nyquist plot in the selected model corresponds to the time constant mainly associated with the R_sl_/CPE_sl_ circuit, i.e., it depends on the surface layer conductivity. The low-frequency (large) arc on the Nyquist plot is associated with the kinetics of the Faraday reaction on the metal, which actually determines the corrosion behavior of steel. The parameters of this semicircle are described by the R_ct_/CPE_dl_ circuit as part of the overall equivalent circuit.

It is evident from the figure that the radii of the arcs on the Nyquist plots of samples treated in CIN vapors are considerably larger than those obtained on steel heat-treated without a CIN. This qualitatively indicates a strong inhibitory effect of the CIN studied. The values of elements obtained by calculations are presented in Table 5.

These results imply that after treatment with a CIN, the surface layer resistance R_sl_ increases by more than an order of magnitude. The resistance of oxide and oxide-inhibitor surface layers is determined by the transfer of anions and cations involved in the corrosion process. Taking the results of the ellipsometric determination of the thicknesses of these layers into account, it is obvious that the layer thickness itself does not correlate with the R_sl_ values obtained. What is more, the highest resistance is obtained for the thinnest layer after treatment with ODA + BTA. Hence, it can be concluded that all the CIN studied drastically hinder ion transport to the active metal surface.

This is also indicated by a sharp decrease in the capacitance of samples (by modulo A of the CPE_sl_ element (Judging by the values of phase factor *n*, all the CPE elements are of a capacitive nature. Although the modulo A formally has the dimension S·s^n^/cm^2^, if phase factor *n* is close to 1, then the parameters of this circuit element can be numerically estimated in capacitance units, F/cm^2^.)). There is no correlation between the thicknesses of oxide and oxide-inhibitor layers and the capacitance of samples. This is probably due to a change in the surface structure and dielectric properties upon treatment with a CIN. It should also be noted that the results of electrochemical impedance spectroscopy, unlike ellipsometry, do not allow one to separate the effect of the oxide layer and the CIN deposited on top of it. 

The phase factor *n*_sl_ lies in the range from 0.85–0.97, which characterizes these systems as rather homogeneous in structure. The closest approximation to an ideal capacitor is obtained upon steel treatment in ODA + BTA vapors.

To complete the discussion of the results concerning the surface layer, let us note that the efficiency of blocking the ion transfer to the active metal increases in the following series: BTA < ODA < ODA + BTA.

Concerning the elements responsible for the low-frequency process that directly affects the corrosion kinetics, it should be noted that after treatment with a CIN, the charge transfer resistance R_ct_ changed most considerably (by a factor of 40–50), whereas the capacitance of the Faraday reaction decreased by only one order of magnitude. Interestingly, the comparison of inhibited samples with each other shows that the charge transfer resistance R_ct_ changes much less strongly (1.36-fold) than the R_sl_ values (3.28-fold). At the same time, the inhibition of the Faraday corrosion process by the CIN changes in the same series: BTA < ODA < ODA + BTA. 

The capacitance of the double electric layer also decreases in this order. It is worth noting that upon the treatment of steel with BTA, the phase factor is n_dl_ = 0.64 (in contrast to the other inhibitors where n = 1). This indicates a significant inhomogeneity and/or diffuseness of the capacitor plates. Recalculation to the parameters of an equivalent ideal capacitor in this chain for comparison with the other results gives 11 × 10^−6^ F/cm^2^.

Thus, BTA has the smallest inhibitory effect among the CIN studied, both in terms of surface blocking and in its effect on the electrochemical process itself. ODA is in second place in this CIN rating based on both parameters. CT of steel with the ODA + BTA formulation manifests the highest inhibiting properties in terms of all parameters. 

The results of experimental data simulation based on an equivalent circuit make it possible to quantitatively estimate the contribution of various mechanisms that provide the inhibiting effect of the CIN and to determine the partial corrosion inhibition coefficients. 

Two principal mechanisms of action of adsorption-type corrosion inhibitors are known, i.e., the blocking and activation mechanisms [26]. In the former case, an inhibitor adsorbs and blocks a fraction of the metal surface, thus, reducing the corrosion rate, but does not alter the kinetics of electrochemical processes on the remaining unblocked surface. In contrast, the activation mechanism implies that corrosion inhibition occurs due to changes in the activation energy of corrosion processes, and hence, their kinetics. Both mechanisms usually operate simultaneously, but their contributions to the inhibitory effect may differ. 

The R_sl_ value in the equivalent circuit that we use reflects the effect of the surface layer and can, therefore, serve as a criterion for estimating the blocking effect of an inhibitor. The coefficient of corrosion inhibition due to surface blocking (γ_sl_) equals the ratio of R_sl_ values of samples after CT and after TT without a CIN:

γ_sl_ = R_sl_^CT^/R_sl_^TT^ (22)

Using a similar approach, the R_ct_ value can be used to estimate the effect of a CIN on the Faraday corrosion process. Hence, the coefficient of electrochemical reaction inhibition by a CIN (γ_ct_) can be determined as the ratio of the charge transfer resistances R_ct_ for inhibited and non-inhibited samples:

γ_ct_ = R_ct_^CT^/R_ct_^TT^ (23)

The degrees of protection by CIN provided by the blocking and activation mechanisms for different variants of steel St3 chamber treatment are shown in Table 6. 

The comparison of γ_sl_ and γ_ct_ values implies a mixed blocking activation mechanism of action of the CIN studied. However, in all the cases, the γ_sl_ < γ_ct_ inequality is observed, which indicates that the activation mechanism is predominant, especially in the case of BTA. 

Thus, on the one hand, the EIS results confirm the conclusions made by the comparison of the CIN efficiency determined in corrosion experiments, and on the other hand, they indicate a mixed blocking activation mechanism of their action. 

### 3.5. Edge Angle Measurement

The formation of adsorption films of the CIN studied resulted in the hydrophobization of the steel surface (Table 7). This follows from the results of measuring the contact angles of surface wetting by distilled water. 

The contact angle *θ* measured on steel samples after one-hour heat treatment at 120 °C without a CIN was 70°. After CT of the metal in BTA vapors, *θ* reached 103°, i.e., additional hydrophobization of the surface occurred. ODA and the mixed CIN are more powerful hydrophobizing agents which provided *θ* = 107° and 110°, respectively. It is important that the *θ* values increased symbatically to the protective properties of the CIN determined in the corrosion and electrochemical tests. 

The data on the increase in *θ* after chamber treatment of steel with the mixed CIN allowed us to anticipate that surface super-hydrophobization would be achieved. To check this assumption, polymodal roughness was created on steel samples before the CT. After treatment of these samples in ODA + BTA vapors, the contact angles *θ* reached values typical of a super-hydrophobic surface. Note that this is the first system known to the authors where steel super-hydrophobicity was achieved by vapor phase treatment of a metal. 

Although there is no unambiguous correlation between the results of accelerated corrosion tests and electrochemical tests, on the one hand, and the total protection time of metals under outdoor conditions, on the other hand, the data given in the previous sections allow us to believe that chamber treatment in ODA + BTA vapors can provide the temporary protection of steel items. The outdoor corrosion tests confirm this conclusion. 

Corrosion damage (speckles) on steel samples that did not undergo chamber treatment appeared after 10 days of exposure to an urban atmosphere under a shelter (Moscow). The adsorption films formed by the mixed CIN provided steel protection for 1.5 months. This result indicates that the mixed CIN can be used, at least, for the inter-operation storage of steel items. 

**Figure 4:**
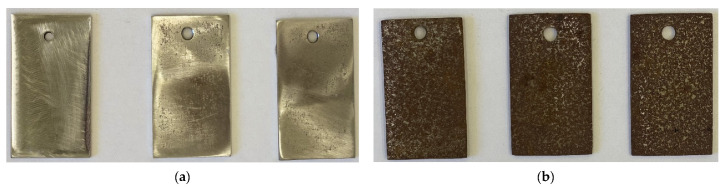
Steel samples after weathering tests for 9 months: chamber treatment with CIN vapors (**a**); without chamber treatment (**b**).

The duration of steel protection with the mixed CIN can be increased significantly by taking additional measures to prevent the deposition of corrosive atmosphere components on the surface of the samples. As such a measure, the samples were packed in an unsealed cardboard box before an outdoor test. In such case, corrosion damage on steel samples without CT appeared after 15 days of their exposure under a shelter, while the protective aftereffect after the formation of adsorption films in ODA + BTA vapors increased to 8 months. The appearance of samples tested for 9 months under the conditions described above is demonstrated in Figure 4.

## 4. Conclusions

1. A CIN comprising the ODA + BTA mixture efficiently protects steel from atmospheric corrosion. It can be used to temporarily protect metal items.
2. The optimum temperature for steel chamber treatment with the BTA + ODA mixed inhibitor is 120 °C. One-hour treatment in vapors of this CIN at this temperature creates nanosized adsorption films that stabilize the metal’s passive state.
3. It has been theoretically shown that in the absence of interactions between the components, the protective properties of the CIN mixture are superior to those of each of its components alone. That said, the synergism between the components is not a prerequisite for the creation of a mixed CIN with a protective effect exceeding that of its components.
4. Despite the fact that the PAE of the ODA + BTA mixture is superior to that of its components, a formal estimation of their mutual effect indicates that an antagonism exists. One of the reasons for the antagonism may be that the vapor pressure of the mixture during CT decreases due to the reaction of the components to form a salt between a weak acid, BTA, and a base, ODA.
5. Chamber treatment of steel in ODA + BTA vapors enhances surface hydrophobization and creates super-hydrophobization conditions on a polymodal metal surface.

## Figures and Tables

**Table 1:** PAE of the adsorption films formed upon steel treatment in CIN vapors at various temperatures. Recurrent moisture condensation conditions. *τ_CE_* = 1 h.

Chamber Inhibitor	Time until the Appearance of Corrosion Damage, h, after Chamber Treatment at Temperature			
	80 °C	100 °C	120 °C	140 °C
None	0.5	1.0	0.5	1.5
BTA	0.5	1.0	3.0	2.0
ODA	1.0	72.0	120.0	48.0
ODA + BTA	1.0	120.0	168.0	144.0

**Table 2:** PAE of the adsorption films formed on steel in CIN vapors at 120 °C and various *τ_CT_*. Recurrent moisture condensation conditions.

Chamber Inhibitor	Time until the Appearance of Corrosion Damage, h, after Chamber Treatment for a Period of			
	20 min	1 h	2 h	4 h
None	0.25	0.5	0.5	0.5
BTA	1.0	1.0	1.0	3.0
ODA	72.0	120.0	120.0	120.0
ODA + BTA	120.0	168.0	168.0	168.0

**Table 3:** Effect of steel CT on the characteristics of anodic potentiodynamic curves. *T*_CT_ = 120 °C, τ_CT_ = 1 h.

Chamber Inhibitor	*E*_cor_, V	*E*_pit_, V	*E*_br_, V
None	0.095	0.150	0.270
BTA	−0.085	0.210	0.210
ODA	−0.130	0.295	0.345
ODA + BTA	0.090	0.360	0.360

**Table 4:** Effect of steel chamber treatment on the thickness *d* of surface films. *T*_CT_ = 120 °C, τ_CT_ = 1 h.

Chamber Inhibitor	Thickness *d*, nm	
	Oxide Film	Adsorbed CIN Film
None	15 ± 0.5	-
BTA	4.5 ± 0.5	4 ± 0.5
ODA	6.5 ± 0.5	6.5 ± 0.5
ODA + BTA	3 ± 0.5	2 ± 0.5

**Table 5:** Impedance parameters. *T*_CT_ = 120 °C, τ_CT_ = 1 h.

Chamber Inhibitor	R_s_, kΩ·cm^2^	R_sl_, kΩ·cm^2^	CPE_sl_A, S·s^n^/cm^2^	CPE_sl_n_sl_	R_ct_ kΩ·cm^2^	CPE_dl_A, S·s^n^/cm^2^	CPE_dl_n_dl_	ProtectionZ, %
None	0.61	11.8	6.75·10^−5^	0.87	10.33	5.46·10^−5^	0.95	-
BTA	0.47	105.73	9.02·10^−7^	0.91	402	6.87·10^−6^	0.67	95.64
ODA	0.59	209.59	9.71·10^−7^	0.85	441	4.27·10^−6^	1	96.58
ODA + BTA	0.84	345.32	6.37·10^−7^	0.97	549	3.37·10^−6^	1	97.53

**Table 6:** Degrees of protection by CIN provided by the blocking and activation mechanisms for different variants of steel St3 chamber treatment.

Chamber Inhibitor	γ_sl_	γ_ct_
BTA	8.9	39.0
ODA	17.8	42.8
ODA + BTA	29.3	53.3

**Table 7:** Effect of CT on the *θ* of steel wetting by distilled water. *T*_CT_ = 120 °C, τ_CT_ = 1 h.

Chamber Inhibitor	Contact Angle, deg.
None	70
BTA	103
ODA	107
ODA + BTA	110
ODA + BTA, polymodal surface	158

## Data Availability

Data sharing is not applicable to this article.

## References

[1] Valdez B., Schorr M., Cheng N., Beltran E., Salinas R. (2018). Technological applications of volatile corrosion inhibitors. Corros. Rev..

[2] Ansari F.A., Verma C., Siddiqui Y.S., Ebenso E.E., Quraishi M.A. (2018). Volatile corrosion inhibitors for ferrous and non-ferrous metals and alloys: A review. Int. J. Corros. Scale Inhib..

[3] Gangopadhyay S., Mahanwar P.A. (2018). Recent developments in the volatile corrosion inhibitor (VCI) coatings for metal: A review. J. Coat. Technol. Res..

[4] Andreev N.N., Kuznetsov Y.I. (2005). Physicochemical aspects of the action of volatile metal corrosion inhibitors. Russ. Chem. Rev..

[5] Bastidas D.M., Cano E., Mora E.M. (2005). Volatile corrosion inhibitors: A review. Anti-Corros. Methods Mater..

[6] Chen Z., Huang L., Zhang G., Qiu Y., Guo X. (2012). Benzotriazole as a volatile corrosion inhibitor during the early stage of copper corrosion under adsorbed thin electrolyte layers. Corros. Sci..

[7] Gelman D., Starosvetsky D., Ein-Eli Y. (2014). Copper corrosion mitigation by binary inhibitor compositions of potassium sorbate and benzotriazole. Corros. Sci..

[8] Focke W.W., Nhlapo N.S., Vuorinen E. (2013). Thermal analysis and FTIR studies of volatile corrosion inhibitor model systems. Corros. Sci..

[9] Zhang D.Q., An Z.X., Pan Q.Y., Gao L.X., Zhou G.D. (2006). Comparative study of bis-piperidiniummethyl-urea and mono-piperidiniummethyl-urea as volatile corrosion inhibitors for mild steel. Corros. Sci..

[10] Pieterse N., Focke W.W., Vuorinen E., Rácz I. (2006). Estimating the gas permeability of commercial volatile corrosion inhibitors at elevated temperatures with thermo-gravimetry. Corros. Sci..

[11] Tsvetkova I.V., Luchkin A.Y., Goncharova O.A., Veselyi S.S., Andreev N.N. (2021). Chamber inhibitors of steel corrosion based on lauric acid. Int. J. Corros. Scale Inhib..

[12] Goncharova O.A., Luchkin A.Y., Archipushkin I.A., Andreev N.N., Kuznetsov Y.I., Vesely S.S. (2019). Vapor-phase protection of steel by inhibitors based on salts of higher carboxylic acids. Int. J. Corros. Scale Inhib..

[13] Goncharova O.A., Luchkin A.Y., Andreev N.N., Andreeva N.P., Vesely S.S. (2018). Triazole derivatives as chamber inhibitors of copper corrosion. Int. J. Corros. Scale Inhib..

[14] Goncharova O.A., Andreev N.N., Luchkin A.Y., Kuznetsov Y.I., Andreeva N.P., Vesely S.S. (2019). Protection of copper by treatment with hot vapours of octadecylamine, 1,2,3-benzotriazole and their mixtures. Mater. Corros..

[15] Luchkin A.Y., Goncharova O.A., Arkhipushkin I.A., Andreev N.N., Kuznetsov Y.I. (2020). The effect of oxide and adsorption layers formed in 5-chlorobenzotriazole vapors on the corrosion resistance of copper. J. Taiwan Inst. Chem. Eng..

[16] Kuznetsov Y., Goncharova O., Luchkin A., Vesely S., Andreev N. Vapor-phase protection of metals from atmospheric corrosion by low-volatile organic inhibitors. Proceedings of the European Corrosion Congress (EUROCORR 2018).

[17] GOST 380-2005 Common Quality Carbon Steel. Grades.

[18] Boinovich L.B., Emelyanenko A.M., Modestov A.D., Domantovsky A.G., Emelyanenko K.A. (2015). Synergistic effect of superhydrophobicity and oxidized layers on corrosion resistance of aluminum alloy surface textured by nanosecond laser treatment. ACS Appl. Mater. Interfaces.

[19] Shcherbakov A.I., Korosteleva I.G., Kasatkina I.V., Kasatkin V.E., Kornienko L.P., Dorofeeva V.N., Vysotskii V.V., Kotenev V.A. (2019). Impedance of an Aluminum Electrode with a Nanoporous Oxide. Prot. Met. Phys. Chem. Surf..

[20] Mansfeld F., Kending M.W., Tsai S. (1982). Recording and Analysis of AC Impedance Data for Corrosion Studies. Corrosion.

[21] Mansfeld F. (1995). Use of electrochemical impedance spectroscopy for the study of corrosion protection by polymer coatings. J. Appl. Electrochem..

[22] Vinogradova S.S., Iskhakova I.O., Kaidrikov R.A., Zhuravlev B.L. (2012). Impedance Spectroscopy Method in Corrosion Studies.

[23] Mahato N., Singh M.M. (2011). Investigation of passive film properties and pittinp resistance of ALSI 316 in aqueous ethanoic acid containing chloride ions using elextrochemical impedance spectroscopy (EIS). Port. Electrochim. Acta.

[24] Macdonald J.R., Barsoukov E. (2005). Impedance spectroscopy: Theory, experiment, and applications. History.

[25] Ekilik V.V., Chikov O.V. (1991). Diagnostic criteria of the mutual effect of inhibitors of acid corrosion of metals. Prot. Met..

[26] Antropov L.I., Makushin V.F., Panasenko V.F. (1981). Metal Corrosion Inhibitors.

[27] Kuznetsov Y.I., Andreev N.N. (1996). Mixed Inhibitors and Some Aspects of Synergism in Corrosion Inhibition; CONF960389.

